# Supplementary material for: Study on molecular orientation and stratification in RNA-lipid nanoparticles by cryogenic orbitrap secondary ion mass spectrometry
Source: Commun Chem. 2025 May 22;8:160. doi: 10.1038/s42004-025-01526-x (PMC12098871; doi:10.1038/s42004-025-01526-x)
Supplement: Supplementary file 2 — Supplementary information [file 42004_2025_1526_MOESM2_ESM.pdf]

Study on molecular orientation and stratification in RNA-lipid nanoparticles by Cryogenic Orbitrap Secondary Ion Mass Spectrometry

Supplementary Information

Figures

a)	Size (nm)	PDI	%EE
LNP Formulation	91.1	0.03	92.5

b)

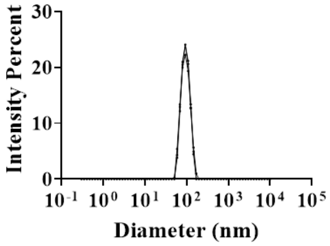

c)

Embedding (ice overlayer):

Supported frozen particles incorporating water:

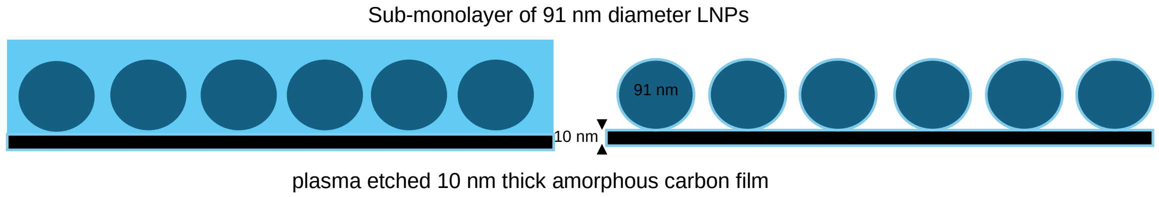

d)

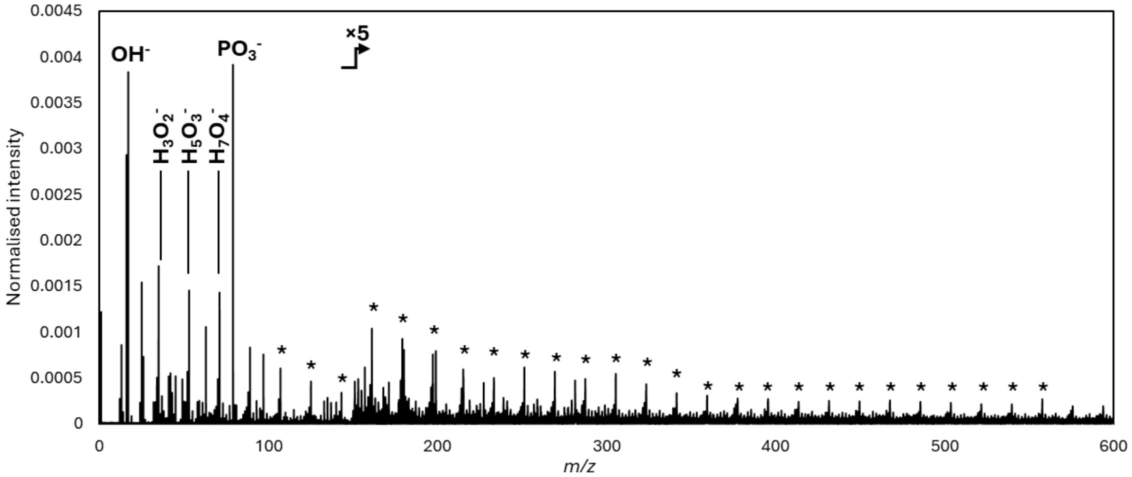

Figure S1 Characterization of LNPs. a) z-average size (nm), PDI, and encapsulation efficiency (%EE) as described in the experimental section. b) DLS size distribution curve. c) Schematic of the two possible frozen nanoparticle situations for the Cryo-OrbiSIMS analysis. The depth profiles do not support the ice embedding option. d) LMIG ToF spectra of the LNP formulation acquired in cryogenic conditions. The repetitive pattern represents water cluster peaks, the first 3 water cluster peaks are assigned in the spectrum, with the other indicated by asterisk.

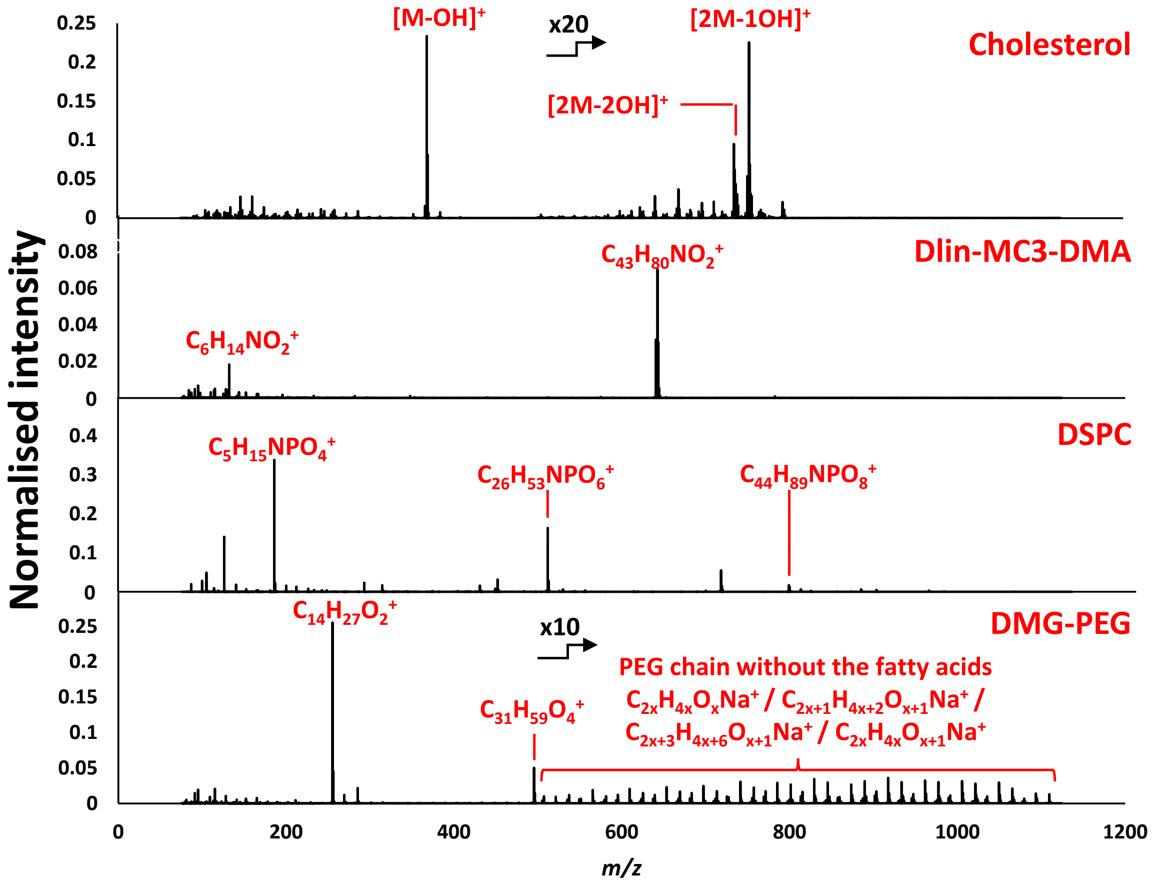

Figure S2 Positive polarity Cryo-OrbiSIMS spectra of individual lipid component samples (*m/z* 75–1125). All spectra are normalised to total ion count and presented on individual intensity scales for clarity.

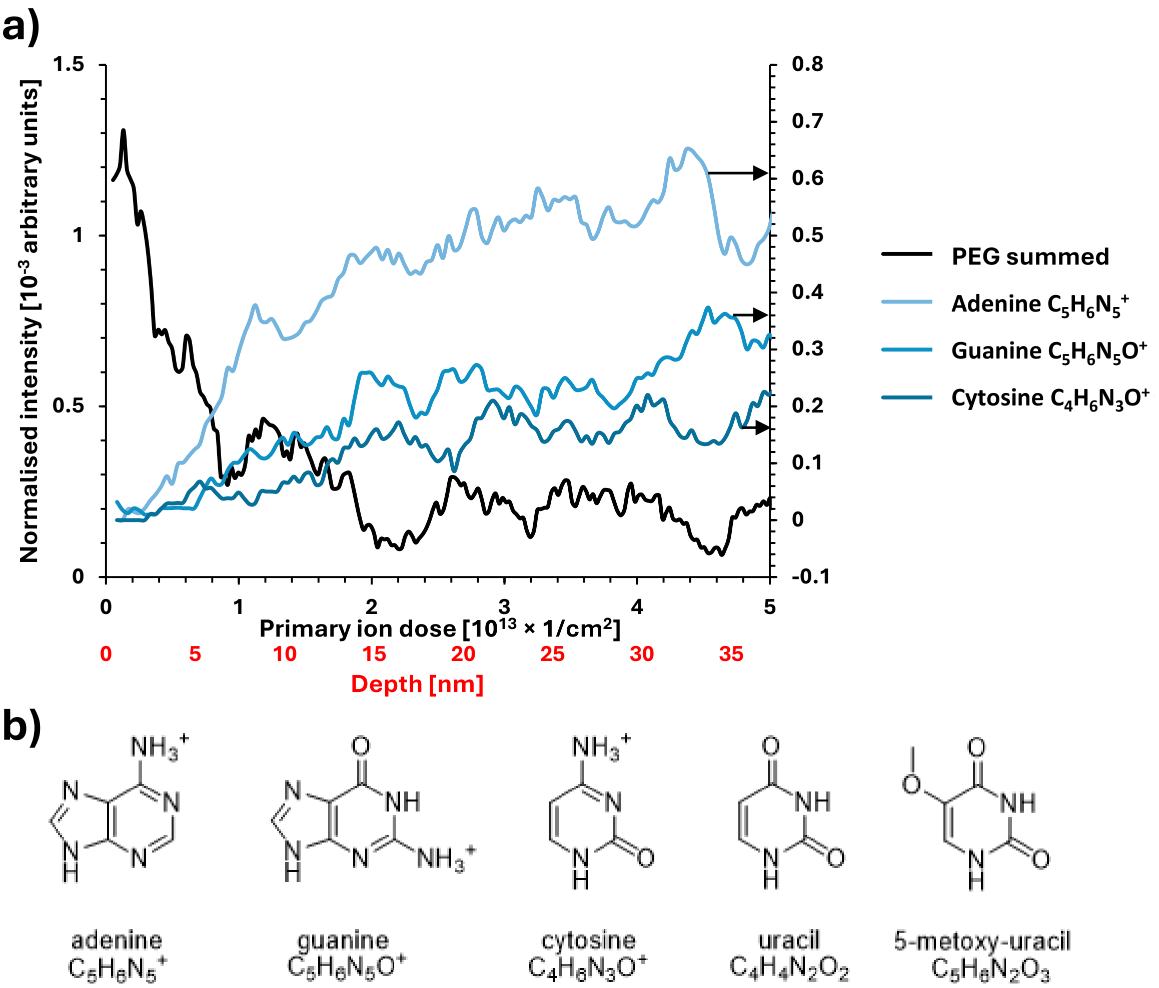

Figure S3 a) Cryo-OrbiSIMS depth profiles of the LNP formulation displaying intensity of RNA fragments: adenine, guanine and cytosine along with the PEG ions. The intensities have been normalised to total ion count and all RNA fragment ions are presented on secondary intensity scale for clarity. Neither an ion representative of 5-methoxyuradine (5-methoxyuracil) or uracil was seen in the spectra. **b)** The detected and missing characteristic ion fragments are from the RNA cargo are listed.

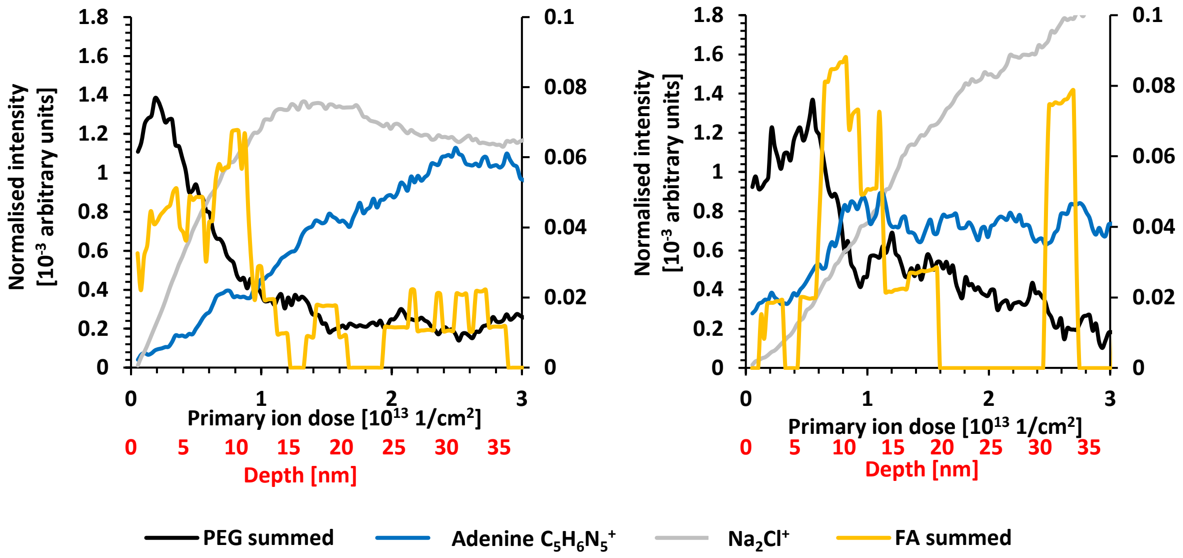

Figure S4 Cryo-OrbiSIMS profiles acquired from on different samples from the same batch of LNPs on different occasions as the data in Figure 1b. The intensity has been normalised to total ion count and the summed FA14:0 ions are presented on secondary intensity scale for both depth profiles for clarity.

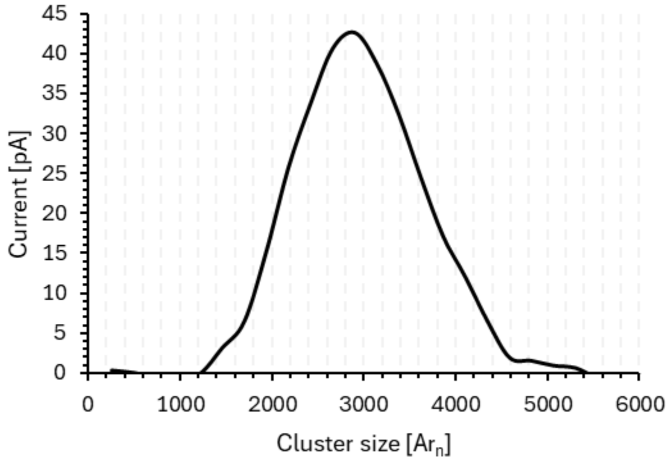

Figure S5 Cluster size distribution of the Ar_{3000}^+ primary ion beam used for Cryo-OrbiSIMS analysis.

Table S1 DMG-PEG2k fragments containing both DMG and PEG fragments. The PEG component, which were summed in the depth profiles.

m/z	SIMS assignment	Atomic composition	Structural formula
227.2016	$C_{14}H_{27}O_2^+$	$H(CH_2)_{13}C(=O)O^+$	$[FA(14:0)]^+$
251.1983	$C_{14}H_{28}O_2Na^+$	$H(CH_2)_{13}C(=O)ONa^+$	$[FA(14:0)]Na^+$
493.2618	$C_{21}H_{42}O_{11}Na^+$	$C_{2x+1}H_{4x+2}O_{x+1}Na^+$	$Na[O\text{-}CH_2\text{-}CH_2]_x\text{-}O\text{-}CH_2^+$
495.4408	$C_{31}H_{59}O_4^+$	DMG^+	$[FA(14:0)]_2C_3H_5^+$
507.2775	$C_{22}H_{44}O_{11}Na^+$	$C_{2x}H_{4x}O_xNa^+$	$Na[O\text{-}CH_2\text{-}CH_2]_x^+$
537.2881	$C_{23}H_{46}O_{12}Na^+$	$C_{2x+1}H_{4x+2}O_{x+1}Na^+$	$Na[O\text{-}CH_2\text{-}CH_2]_x\text{-}O\text{-}CH_2^+$
551.3037	$C_{24}H_{48}O_{12}Na^+$	$C_{2x}H_{4x}O_xNa^+$	$Na[O\text{-}CH_2\text{-}CH_2]_x^+$
565.3193	$C_{25}H_{50}O_{12}Na^+$	$C_{2x+3}H_{4x+6}O_{x+1}Na^+$	$NaCH_2[O\text{-}CH_2\text{-}CH_2]_x\text{-}O\text{-}CH_2\text{-}CH_2^+$
567.2985	$C_{24}H_{48}O_{13}Na^+$	$C_{2x}H_{4x}O_{x+1}Na^+$	$Na[O\text{-}CH_2\text{-}CH_2]_xO^+$
581.3142	$C_{25}H_{50}O_{13}Na^+$	$C_{2x+1}H_{4x+2}O_{x+1}Na^+$	$Na[O\text{-}CH_2\text{-}CH_2]_x\text{-}O\text{-}CH_2^+$
609.3454	$C_{27}H_{54}O_{13}Na^+$	$C_{2x+3}H_{4x+6}O_{x+1}Na^+$	$NaCH_2[O\text{-}CH_2\text{-}CH_2]_x\text{-}O\text{-}CH_2\text{-}CH_2^+$
625.3403	$C_{27}H_{54}O_{14}Na^+$	$C_{2x+1}H_{4x+2}O_{x+1}Na^+$	$Na[O\text{-}CH_2\text{-}CH_2]_x\text{-}O\text{-}CH_2^+$
653.3717	$C_{29}H_{58}O_{14}Na^+$	$C_{2x+3}H_{4x+6}O_{x+1}Na^+$	$NaCH_2[O\text{-}CH_2\text{-}CH_2]_x\text{-}O\text{-}CH_2\text{-}CH_2^+$
669.3665	$C_{29}H_{58}O_{15}Na^+$	$C_{2x+1}H_{4x+2}O_{x+1}Na^+$	$Na[O\text{-}CH_2\text{-}CH_2]_x\text{-}O\text{-}CH_2^+$
697.3978	$C_{31}H_{62}O_{15}Na^+$	$C_{2x+3}H_{4x+6}O_{x+1}Na^+$	$NaCH_2[O\text{-}CH_2\text{-}CH_2]_x\text{-}O\text{-}CH_2\text{-}CH_2^+$
713.3928	$C_{31}H_{62}O_{16}Na^+$	$C_{2x+1}H_{4x+2}O_{x+1}Na^+$	$Na[O\text{-}CH_2\text{-}CH_2]_x\text{-}O\text{-}CH_2^+$
741.424	$C_{33}H_{66}O_{16}Na^+$	$C_{2x+3}H_{4x+6}O_{x+1}Na^+$	$NaCH_2[O\text{-}CH_2\text{-}CH_2]_x\text{-}O\text{-}CH_2\text{-}CH_2^+$
757.4189	$C_{33}H_{66}O_{17}Na^+$	$C_{2x+1}H_{4x+2}O_{x+1}Na^+$	$Na[O\text{-}CH_2\text{-}CH_2]_x\text{-}O\text{-}CH_2^+$
785.4502	$C_{35}H_{70}O_{17}Na^+$	$C_{2x+3}H_{4x+6}O_{x+1}Na^+$	$NaCH_2[O\text{-}CH_2\text{-}CH_2]_x\text{-}O\text{-}CH_2\text{-}CH_2^+$
801.4452	$C_{35}H_{70}O_{18}Na^+$	$C_{2x+1}H_{4x+2}O_{x+1}Na^+$	$Na[O\text{-}CH_2\text{-}CH_2]_x\text{-}O\text{-}CH_2^+$
829.4766	$C_{37}H_{74}O_{18}Na^+$	$C_{2x+3}H_{4x+6}O_{x+1}Na^+$	$NaCH_2[O\text{-}CH_2\text{-}CH_2]_x\text{-}O\text{-}CH_2\text{-}CH_2^+$
845.4713	$C_{37}H_{74}O_{19}Na^+$	$C_{2x+1}H_{4x+2}O_{x+1}Na^+$	$Na[O\text{-}CH_2\text{-}CH_2]_x\text{-}O\text{-}CH_2^+$
873.5026	$C_{39}H_{78}O_{19}Na^+$	$C_{2x+3}H_{4x+6}O_{x+1}Na^+$	$NaCH_2[O\text{-}CH_2\text{-}CH_2]_x\text{-}O\text{-}CH_2\text{-}CH_2^+$
889.4975	$C_{39}H_{78}O_{20}Na^+$	$C_{2x+1}H_{4x+2}O_{x+1}Na^+$	$Na[O\text{-}CH_2\text{-}CH_2]_x\text{-}O\text{-}CH_2^+$
917.5291	$C_{41}H_{82}O_{20}Na^+$	$C_{2x+3}H_{4x+6}O_{x+1}Na^+$	$NaCH_2[O\text{-}CH_2\text{-}CH_2]_x\text{-}O\text{-}CH_2\text{-}CH_2^+$
933.5238	$C_{41}H_{82}O_{21}Na^+$	$C_{2x+1}H_{4x+2}O_{x+1}Na^+$	$Na[O\text{-}CH_2\text{-}CH_2]_x\text{-}O\text{-}CH_2^+$
961.5549	$C_{43}H_{86}O_{21}Na^+$	$C_{2x+3}H_{4x+6}O_{x+1}Na^+$	$NaCH_2[O\text{-}CH_2\text{-}CH_2]_x\text{-}O\text{-}CH_2\text{-}CH_2^+$
977.5498	$C_{43}H_{86}O_{22}Na^+$	$C_{2x+1}H_{4x+2}O_{x+1}Na^+$	$Na[O\text{-}CH_2\text{-}CH_2]_x\text{-}O\text{-}CH_2^+$
1005.581	$C_{45}H_{90}O_{22}Na^+$	$C_{2x+3}H_{4x+6}O_{x+1}Na^+$	$NaCH_2[O\text{-}CH_2\text{-}CH_2]_x\text{-}O\text{-}CH_2\text{-}CH_2^+$
1021.576	$C_{45}H_{90}O_{23}Na^+$	$C_{2x+1}H_{4x+2}O_{x+1}Na^+$	$Na[O\text{-}CH_2\text{-}CH_2]_x\text{-}O\text{-}CH_2^+$
1049.608	$C_{47}H_{94}O_{23}Na^+$	$C_{2x+3}H_{4x+6}O_{x+1}Na^+$	$NaCH_2[O\text{-}CH_2\text{-}CH_2]_x\text{-}O\text{-}CH_2\text{-}CH_2^+$
1065.602	$C_{47}H_{94}O_{24}Na^+$	$C_{2x+1}H_{4x+2}O_{x+1}Na^+$	$Na[O\text{-}CH_2\text{-}CH_2]_x\text{-}O\text{-}CH_2^+$

1093.633	$C_{49}H_{98}O_{24}Na^+$	$C_{2x+3}H_{4x+6}O_{x+1}Na^+$	$NaCH_2[O\text{-}CH_2\text{-}CH_2]_x\text{-}O\text{-}CH_2\text{-}CH_2^+$
1109.628	$C_{49}H_{98}O_{25}Na^+$	$C_{2x+1}H_{4x+2}O_{x+1}Na^+$	$Na[O\text{-}CH_2\text{-}CH_2]_x\text{-}O\text{-}CH_2^+$
